# Blood Cure of Chronic Gastric Catarrh

**Published:** 1900-11

**Authors:** T. J. Biggs

**Affiliations:** Stamford, Conn.


### BLOOD CURE OF CHRONIC GASTRIC CATARRH.

By T. J. BIGGS, M. D., Stamford, Conn.

SAMUEL A., aged 34 years, English; admitted to hospital June 2, 1900. Diagnosis: chronic gastric catarrh. The case was sent to me by Dr. R., he having given up all hope of doing anything for it himself. Prior to being treated by Dr. R., the case had been in St. Luke's Hospital for six months, but had received little or no benefit. The general symptoms presenting were: loss of appetite, disagreeable gnawing, and, at times, fulness in the stomach; tenderness at the epigastrium, slightly influenced by eating; almost constant prominence of the epigastrium from distension by decomposing gases. The patient has occasional attacks of nausea and vomiting, occurring most frequently on arising, consisting of gray mucus, raised after great retching; constant thirst; often great burning at the pit of the stomach; bowels constipated; urine high colored. There was a constant feeling of mental depression, and sleeplessness, with occasional attacks of vertigo. The patient also had a follicular pharyngitis of an aggravated type. He was very thin, muscles relaxed, and the skin dry. On entering the hospital he was so weak that he had to be carried from the ambulance on a litter.

His secretions were regulated and he was put on an absolute bovinine diet, half a teaspoonful every hour in lime water and peptonised milk. Once in twenty-four hours he was rubbed thoroughly with olive oil. The follicular pharyngitis was first treated by cleansing the surface with bovinine-Thiersch, followed by spraying the bovinine pure, this being employed every three hours.

On June 10th, the patient felt stronger, was sleeping well, and was not so depressed mentally, burning in the pit of the stomach greatly reduced, no vomiting, but still present nausea and constant thirst. The bovinine was now increased to a tablespoonful every two hours, and the treatment of the pharyngitis reduced to twice in 24 hours. June 20, the patient was up and about, feeling much stronger, having gained five pounds in weight. The constant thirst had disappeared, as well as the nausea. He also craved some general diet. He was, however, perfectly nourished and did not complain of being hungry, only thought he would like to try and eat something. This was not as yet allowed. June 22, however, his condition still being on the gain, he was allowed a little rare beef, well chopped, and a piece of toast. This he ate with relish, and retained it without any discomfort. Treatment continued.

June 23, the follicular pharyngitis had entirely disappeared. He was allowed some rare chopped beef, a little rice and toast. June 25, he took a long walk and on returning, said he felt splendidly. On the 28th, he was discharged cured, with the advice to continue the bovinine and to report for examination at the end of a week.

The action of the bovinine in this class of cases, as in all others of a similar kind, is: it gives the alimentary tract absolute rest, and at the same time supplies perfect nutrition, containing as it does adequate elements in the proper proportion to sustain the human organism.

